# Impact of P2Y_12_-mediated platelet reactivity on myocardial perfusion of patients with ST-segment elevation myocardial infarction undergoing primary percutaneous coronary intervention: a cardiac magnetic resonance study

**DOI:** 10.1186/1532-429X-17-S1-P152

**Published:** 2015-02-03

**Authors:** Alessio La Manna, Piera Capranzano, Chiara Bucciarelli-Ducci, Antonella Salemi, Irene Cascone, Alessandra Cadoni, Claudia I Tamburino, Davide Capodanno, Corrado Tamburino

**Affiliations:** Cardiology Division, Ferrarotto Hospital, University of Catania, Catania, Italy; Bristol Heart Institute, University of Bristol, Bristol, UK

## Background

Whether high platelet reactivity (HPR) at the time of angiography is associated with worse myocardial reperfusion after primary percutaneous coronary intervention (PPCI) for ST-segment elevation myocardial infarction (STEMI) is unknown. This study aimed to assess the impact of HPR on infarct size and reperfusion injury determined by cardiac magnetic resonance (CMR) in patients with STEMI undergoing PPCI.

## Methods

Patients with STEMI undergoing PPCI and pretreated with a P2Y_12_-receptor antagonist (clopidogrel, prasugrel or ticagrelor) underwent platelet function testing at the time of angiography and a CMR from 7 to 10 days after the index event. Platelet function testing was performed with the VerifyNow assay. HPR was defined according to expert consensus definitions. Central core laboratory-masked analyses for quantified ventricular function, volumes, infarct size, area at risk (edema), microvascular obstruction, intramyocardial hemorrhage were performed. These latter parameters were compared between HPR and non-HPR patients.

## Results

**Figure 1:**
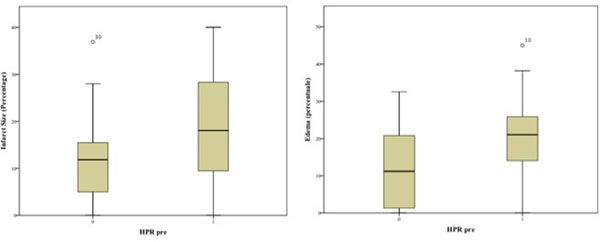
Figure 1

A total of 36 patients were included in the analysis, including 20 (55.5%) with HPR and 16 (44.4%) with non-HPR. No relevant significant differences between HPR and no-HPR groups were found for clinical characteristics, procedural variables, and time intervals. Compared with non-HPR patients, those with HPR had a significantly higher area at risk (p=0.04) and a trend towards higher infact size (p=0.07) (Figure 1). The microvascular obstruction was observed in overall 5 cases (13.9%): 4 (20%) with HPR e in 1 (6.3%) with non-HPR. The intramyocardial hemorrhage was found in 2 patients: one with HPR, but receiving a glycoprotein IIb/IIIa inhibitor in bailout, and one with non-HPR.

## Conclusions

In patients with STEMI undergoing PPCI pretreated with a P2Y_12_ receptor antagonist, the presence of HPR at the time of angiography was associated with higher area at risk and infarct size. These findings suggest the need for achieving at earliest as possible an effective P2Y_12_-inhibition.

## Funding

None.

